# The Endocrine Milieu and CD4 T-Lymphocyte Polarization during Pregnancy

**DOI:** 10.3389/fendo.2014.00106

**Published:** 2014-07-07

**Authors:** Barbara Polese, Virginie Gridelet, Eleni Araklioti, Henri Martens, Sophie Perrier d’Hauterive, Vincent Geenen

**Affiliations:** ^1^GIGA-I3, Center of Immunoendocrinology, University of Liège, Liège, Belgium

**Keywords:** progesterone, estradiol, human chorionic gonadotropin, luteinizing hormone, regulatory T cells, T helper 17 cells, pregnancy

## Abstract

Acceptance of the fetal semi-allograft by the mother’s immune system has become the focus of intensive research. CD4+ T cells are important actors in the establishment of pregnancy. Th1/Th2 paradigm has been expanded to include CD4+ regulatory T (Treg) and T helper 17 (Th17) cells. Pregnancy hormones exert very significant modulatory properties on the maternal immune system. In this review, we describe mechanisms by which the endocrine milieu modulates CD4 T cell polarization during pregnancy. We first focused on Treg and Th17 cells and on their importance for pregnancy. Secondly, we review the effects of pregnancy hormones [progesterone (P4) and estradiol (E2)] on immune cells previously described, with a particular attention to human chorionic gonadotropin (hCG). The importance of Treg cells for pregnancy is evidenced. They are recruited before implantation and are essential for pregnancy maintenance. Decreased number or less efficient Treg cells are implicated in fertility disorders. As for Th17 cells, the few available studies suggest that they have a negative impact on fertility. Th17 frequency is increased in infertile patients. With the combination of its pro-effects on Th2 and Treg cells and anti-effects on Th1 and Th17 cells, P4 contributes to establishment of a favorable environment for pregnancy. E2 effects are more dependent on the context but it seems that E2 promotes Treg and Th2 cells while it inhibits Th1 cells. hCG positively influences activities of Treg and uterine natural killer cells. This embryo signal is an essential actor for the success of pregnancy, both as the endocrine factor regulating P4 secretion by the ovarian corpus luteum, but also as a paracrine agent during implantation as well as an angiogenic and immunologic mediator during the course of gestation. Luteinizing hormone (LH) immune properties begin to be studied but its positive impact on Treg cells suggests that LH could be a considerable immunomodulator in the mouse.

## Introduction

Pregnancy constitutes an immunological paradox since it implies that a fetus semi-antigenically distinct from the mother is not rejected by her immune system from embryo implantation to delivery. Peter Medawar was the first to consider the fetus as a semi-allograft and to suggest a major role for the immune system in ensuring maintenance of pregnancy (1). Since then, the establishment of tolerance of mother’s immune system to the embryonic and fetal semi-allograft has become the focus of intensive research.

Uterine Natural Killer (uNK) cells have been demonstrated to be main actors of pregnancy with their effects on angiogenesis, vascular remodeling, trophoblast invasion, and cytokine production (2, 3). CD4+ T cells are also important actors in the establishment of a «pregnancy favorable environment», and the Th1/Th2 paradigm has been prevailing for years. Briefly, feto-maternal acceptance was explained by Th2 profile predominance essential for pregnancy while pro-inflammatory Th1 cytokines were shown to be downregulated (4). This Th1/Th2 paradigm has been expanded to include CD4+ regulatory T (Treg) cells and interleukin-17 (IL-17) expressing T [T helper 17 (Th17)] cells since it appeared that some studies were not fitting with the original theory (5, 6).

The endocrine system is also essential for the programing of a tolerogenic environment favorable to embryo implantation and fetal development, in particular the pregnancy hormones progesterone (P4), estradiol (E2), and human chorionic gonadotropin (hCG) hormone. Actually, both endocrine and immune systems are intimately linked and pregnancy hormones exert very significant modulatory properties on the maternal immune system.

Here, we review the mechanisms by which the endocrine milieu modulates CD4 T-lymphocyte polarization during pregnancy. First, we will focus on Treg and Th17 cells, on their importance for pregnancy, as well as their implication in infertility disorders. Second, we will review the effects of pregnancy hormones on immune cells previously described, with a particular attention to hCG.

## CD4 T Cells Important for Pregnancy

### Th1 and Th2 cells

**Figure 1:**
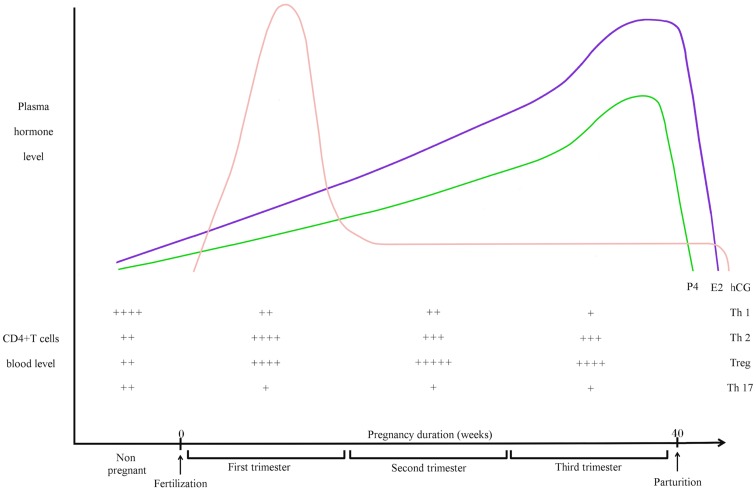
**Pregnancy hormones and CD4+ T cells blood levels during human pregnancy**. hCG is an embryonic signal that announces the presence of an embryo to the maternal organism. *hCG* gene is transcribed as early as the eight-cells stage but it cannot be detected in the maternal blood before the second week after fertilization. Blood hCG concentration reaches a peak between the 8th and 11th week then it declines and stays low until the end of pregnancy. E2 is mainly produced by ovarian granulosa cells and by placenta. E2 concentration in maternal blood increases gradually after the first week, reaches a peak before parturition then drops few days after. Secreted mainly by corpus luteum then by placenta after 12 weeks of pregnancy, small quantities of P4 are already produced at the follicle stage. Then, P4 concentration in the blood increases strongly during the course of pregnancy to reach a peak before parturition. Relative blood levels of CD4+ T cells are compiled from different articles. During healthy pregnancy, Th1 cells are downregulated while Th2 cells are upregulated (13). Th2 predominance is considered to be essential for fetal survival. Pregnancy is also associated with a systemic expansion of Treg cells. The level of Treg cells is on the peak during the second trimester (14, 15). The circulating level of Th17 cell does not vary during human pregnancy and stays low (16). They are decreased during pregnancy when compared to non-pregnant women (17).

CD4+ T cells are heterogenous members of the adaptative immune system. Different subsets have been identified based on their distinct cytokine and transcriptional profiles. Each subset has different effector functions. To be short, Th1 cells are polarized by IL-12 and are characterized by high production of interferon-gamma (IFNγ). Th2 cells are polarized mainly by IL-4 and produce a cytokine profile including IL-4, IL-5, IL-6, and IL-13. Th2 are important for the clearing of extra-cellular pathogens and for helping B cells to produce antibodies. A balance between Th1 and Th2 is important for immune response, and Th1 and Th2 differentiation is mutually exclusive (7). In the context of pregnancy, Th1/Th2 balance was seen as essential for determining fetus survival in the maternal uterus. Th2 predominance was considered to be essential for fetal survival while a polarized Th1 profile could promote fetal rejection (4, 8–11). Th1/Th2 proportions during human pregnancy are simplified in Figure 1. However, the Th1/Th2 paradigm has been challenged by some recent studies that, for example, showed that Th2 cytokines KO mice (IL-4/IL-10 double KO mice) had no fertility disorders (12). For a complete review about the questioning of Th1/Th2 paradigm, please refer to the comprehensive review of Chaouat (5).

### Treg cells

During the 1990s, Sakaguchi identified a T cell subpopulation naturally present in the immune system indispensable for tolerance and immune homeostasis. Those cells specialized in suppression/regulation of the immune response were called Treg cells (18) that were characterized first by increased CD25 expression at their surface (18). In 2003, Foxp3 was then discovered as the specific transcription factor that induces Treg cell differentiation (19). Treg cells are phenotypically and functionally heterogenous. Currently, various subsets of Treg cells in immune system have been identified (20). One common classification distinguishes thymic Treg (tTreg) generated in the thymus by a selection process from peripheral Treg or inducible Treg (iTreg) developing in the periphery from naïve T cells after antigenic stimulation. It has been shown that CNS1 at the *Foxp3* locus is not required for tTreg differentiation while it is essential for iTreg generation (21). The Treg cell family includes also IL-10-secreting CD4+ T regulatory-1 cells (Tr1) and TGF-β-secreting CD4+ Th cells (Th3). Treg cells exert their suppressive functions by different mechanisms such as secretion of inhibitory cytokines, cytolysis or metabolic disruption of the target cell and modulation of antigen presentation (20). As major actors for immunological tolerance, their impact on the acceptance of the fetus by the mother has been explored.

It is now more and more demonstrated that Treg cells are important cells for embryo implantation and pregnancy. Aluvihare et al. were the first to demonstrate that Treg cells mediate maternal tolerance to the fetus in the mouse (22). They observed a systemic expansion of the maternal CD4+CD25+ T cell pool during pregnancy and expression of Foxp3 by uterine CD4+CD25+ cells. They demonstrated also that CD25+ T cells depletion leads to gestation failure. Two studies confirmed this important role of Treg cells in human pregnancy (14, 23). Figure 1 depicts Treg cell blood levels during human pregnancy. Later, Zenclussen and her colleagues observed that a decrease in Treg cell activity leads to spontaneous abortion and they also demonstrated that adoptive transfer of Treg cells is able to prevent fetal rejection in a murine abortive model (24). They showed afterwards that the transferred Treg cells act by creating a privileged tolerant environment, and by up-regulating leukemia inhibitory factor (LIF), TGF-β, and HO-1 levels (25). Tilburgs et al. reported selective migration of specific Treg cells from blood to the decidua in human pregnancy (26). Then, seminal fluid was shown to drive expansion of CD4+CD25+ cells in mouse (27). By injecting anti-CD25 mAb in pregnant mice at different time of pregnancy, Shima et al. demonstrated that Treg cells are necessary for implantation and maintenance of early pregnancy but not late pregnancy in allogeneic mice (28). Over the years, the in-depth study of Treg cells gave rise to the question of which subtype of Tregs is really acting during pregnancy (29). Samstein et al. studied this issue for the first time. They demonstrated that iTregs play important role in maintenance of pregnancy by using CNS1-deficient female. Those females present an increased frequency in fetal resorption (30). Zenclussen and her team were also interested in this subject. By using Helios as a marker of tTreg cells (31), they showed that tTreg cells are important for mouse pregnancy establishment while iTregs act at later pregnancy stages (32). Recently, Rowe et al. showed in mice that specific fetal Treg cells persist after delivery and re-accumulate during subsequent pregnancy in order to sustain protective regulatory memory to fetal antigen (33). The study of memory Treg cells across pregnancy remains another important topic to explore in the near future.

In addition, some papers evidenced a Treg cell dysregulation in infertile patients. A decrease in the decidual Treg cell number has been reported in human miscarriage (23). Endometrial expression of *Foxp3* mRNA is decreased in women with primary unexplained infertility (34). Treg frequency in women with recurrent pregnancy lost do not fluctuate during menstrual cycle in comparison with fertile women, but Treg cell function in those women is deficient (35). The suppressor activity of iTregs in those patients is also decreased (36). Regarding preeclampsia (PE), it has been reported that PE patients present decreased Treg cell level in blood and in decidua (17, 37, 38). Moreover, Treg cell function has been found to be decreased in PE patients (39). Recently, Inada et al. showed that the population of decidual non-proliferating Foxp3+ Tregs is significantly smaller in cases of miscarriages than in normal pregnancy (40). This suggests that non-proliferating Treg cells are important for induction of immune tolerance. Finally, in the CBA × DBA/2 well-known mouse model of abortion (41), a decreased number in Treg cells has also been reported (24). Altogether, those studies demonstrate that abnormalities in Treg cell frequency and function exert a negative impact upon fertility and confirm their importance for pregnancy.

### Th17 cells

T helper 17 cells are a lineage of CD4+ T cells characterized by the secretion of IL-17a and IL-17f cytokines (42, 43) and by the expression of RORc (RORγt in mice) specific transcription factor (44). TGF-β, IL-6, IL-21, and IL-23 are important for their differentiation. It has to be mentioned that Th17 differentiation in mice is quite different from this process in humans (45). By the secretion of IL-17 cytokines, Th17 trigger recruitment, activation, and migration of neutrophils but also liberation of pro-inflammatory mediators. Th17 cells produce other cytokines too. IL-21 is involved in expansion of activated B cells and in class switching of immunoglobulin isotypes. The secretion of IL-21 can also contribute to an autoamplification loop of Th17 cells by inducing their own differentiation. As for IL-22 production, it impacts on epithelial and endothelial barrier function through a dual effect on inflammation and tissue repair induction (46). Thus, Th17 cells are important effector cells for defense against extra-cellular pathogens. They are also associated with the pathogenesis of several autoimmune and inflammatory diseases (45). This complexity of Th17 cells is further amplified by their plasticity and instability. They can acquire other phenotypes depending on the cytokine environment (47). Giving the increasing demonstration of Th17 cell importance in the homeostasis of the immune system, their influence during pregnancy began to be investigated in the past few years.

Santner-Nanan et al. first found a lower Th17 cell frequency during pregnancy compared with non-pregnant women (17). Another article showed that the circulating level of Th17 cells does not vary during human pregnancy (see Figure 1) and that decidual levels of Th17 cells are higher than in peripheral blood (16). On the contrary, Mjosberg et al. found fewer Th17 cells in human decidua than in blood in early pregnancy and highlighted their nearly absence in decidua (48). Recently, it has been shown that NK cells promote tolerance by dampening Th17 cells via IFNγ at materno-fetal interface, in humans and mice (49). All those results suggest that pregnancy is associated with a decrease in Th17 frequency. This is in agreement with another paper showing that placental trophoblasts in culture with T lymphocytes inhibit Th17 cells while promoting Th2 (50). Actually, not much is known about Th17 physiology during normal pregnancy, and most of articles focus on their presence in infertile patients.

The proportion of Th17 cells has been shown to be higher in blood and decidua of patients with unexplained recurrent spontaneous abortion (51, 52). Th17 factors like RORc and IL-17 are also higher in deciduas of those women. Nakashima et al. found also a high number of IL-17 positive cells in decidua of abortion case and suggested that Th17 cells could be involved in induction of inflammation in the late stage of abortion (53). Ito et al. suggested that Th17 cells could promote inflammation at the feto-maternal interface in preterm delivery, a disorder associated with infection and uterine inflammation (54). Furthermore, it was shown that circulating Th17 cells are increased in PE patients compared with healthy pregnant women (17). Others confirmed those results (39, 55). In a recent and well-thought article, it has been shown that NK cell-mediated inhibition of Th17 is lost in patients with recurrent spontaneous abortion, leading to a Th17 response and inflammation (49). Elevated IL-17 levels have also been observed in plasma of patients suffering from unexplained infertility (56). Altogether, those results indicate that Th17 cells seem to exert a negative impact upon fertility. Not much is known about their physiological role during estrous cycle and pregnancy but their presence is associated with inflammation and infertility. Given the importance of some temporary inflammation state for embryo implantation (57), it would be interesting to better study their exact importance during estrous cycle and early normal pregnancy.

## Immunomodulatory Properties of Pregnancy Hormones

### Progesterone

Progesterone is a crucial hormone in the female reproductive system. Secreted by the ovarian corpus luteum (CL) then by placenta after 12 weeks of pregnancy, it regulates endometrium modifications across the cycle and decidualization in order to prepare the uterus for embryo implantation. P4 plasma levels during human pregnancy are illustrated on Figure 1. Most importantly, P4 has a major impact on establishment and maintenance of pregnancy. Besides its endocrine effects, P4 has immunological properties. In particular, its immunosuppressive effects are known for a long time (58).

In 1995, Piccinni et al. demonstrated that P4 favors the development of Th2 CD4+ T cells (59). They suggested that P4 could be responsible at least in part for the Th2 predominance of pregnancy. A few years later, it was shown that P2 acts via an immunomodulatory protein called P4-inducible blocking factor (PIBF) that increased the production of Th2 cytokines by mice lymphocytes (60). This pro-Th2 effect of P4 is consistent with the increased IL-4 production observed during luteal phase of the ovarian cycle that is associated with elevated levels of P4 and estrogens (61). Moreover, P4 directly inhibits Th1 development while enhancing Th2 polarity in mouse cells (62), and P4 down-regulates the expression of IFN-γ during luteal phase (63). The emergence of the Th1/Th2/Treg/Th17 paradigm led to the study of P4 on those cells too. Mjosberg et al. pointed out the P4 regulatory role on Treg cells during human pregnancy (64). *In vivo* and *in vitro* models indicate that P4 increases the proportion of Treg cells but also enhances their suppressive capacity (65). The association between Treg and P4 levels was confirmed in humans (66) and Lee et al. showed that P4 promotes the differentiation of human cord blood fetal T cells into Treg cells (67). P4 also promotes generation of iTreg cells that are highly stable (68), while it suppresses murine Th17 cells. In human and murine T cells, P4 inhibits differentiation of Th17 and decreases associated factors like RORc and IL-17a. Recently, it was shown that P4 inhibits Th17 response while enhancing Treg development in murine vaginal gonococcal infection (69). Some researchers were curious to know how P4 acts on T cells. Membrane P4 receptors are found in human T cells (70). On the other hand, Treg cell expansion has been suggested to involve nuclear P4 receptors (65). Finally, Lee suggested that both nuclear and non-nuclear receptors are concerned (68). PIBF is one of the target genes of P4 in pregnancy lymphocytes. It signals via Jak1/Stat6 pathway to regulate cytokines expression (71, 72). Altogether, those studies suggest that P4 favors Th2 and Treg cells whilst dampening Th1 and Th17. Thus, P4 seems to participate in establishment of favorable environment for pregnancy by its effects on T cells.

### Estrogens

Estrogens are also important for female reproductive tract, namely E2, estrone (E1), and estriol (E3). E2 is mainly produced by ovarian granulosa cells and by placenta. Figure 1 illustrates E2 plasma levels during human pregnancy. E2 immunomodulatory roles are multiple (73) but there is a paradox between their pro- and anti-inflammatory effects. Particularly, modifications in the clinical state of autoimmune diseases states observed in pregnant patients are distinct when looking at different kinds of diseases. For example, a remission of rheumatoid arthritis (RA) is usually observed in pregnant women (74) while a worsening of symptoms can be noticed during pregnancy for women suffering from systemic lupus erythematosus (SLE) (75). Those observations could be explained by the distinct effects of E2 on immune cell types.

Estradiol receptors (ER) α and β are nuclear receptors expressed on most immune cells (76), including human and murine lymphocytes (77–80). Human CD4+ T cell express ERα at higher levels than B cells (81). These observations led to the study of the immunological properties of E2. E2 up-regulates IFN-γ levels in murine splenic lymphocytes (82) and, in non-obese diabetic (NOD) mice, E2 increase IFN-**γ** production by CD4+ Th1 cells (83). Maret has shown that E2 promotes Th1 cells responses and that requires ERα (84). E2 modulates cytokines and chemokines expression by human and murine dendritic cells (85, 86), which influences secondarily T cell response. E2 acts also on CCR expression and function in murine T lymphocytes (87). In addition to pro-Th1 effects, E2 acts on Th2 cells. Indeed, the increase of IL-4 observed during the luteal phase (corresponding to P2 and E2 high levels) could be caused also by E2 (61). E2 also increases secretion of IL-4 by CD4+ T cells as well as GATA-3 expression in mice (88). Anti- and pro-inflammatory effects have been reported for E2 since it enhances both IL-10 and IFN-**γ** secretion in humans (89). So, E2 would have stimulating effects both on Th2 and Th1. With regard to the Th1/Th2 paradigm of pregnancy, both pro-Th1 and Th2 roles are paradoxical. Doria and others have explained this paradox by stating that, with high E2 levels such as in pregnancy, Th1 development is inhibited while Th2 polarity is favored. This is consistent with the improvement of Th1-mediated diseases and the worsening of Th2-mediated diseases observed during pregnancy (90). On the contrary, by promoting Th2 responses, E2 tends to worsen Th2-mediated diseases like SLE. Study of the E2 impact upon Treg cells has been widely explored, clarifying the situation. First, Polanczyk et al. discovered that E2 enhances Foxp3 expression *in vivo* and *in vitro* (91). They suggested for the first time that E2 helps to regulate fetal tolerance during pregnancy by expanding Treg cells. One year later, the same team showed that E2 also increases the suppressive function of Treg cells. Similarities between pregnant mice and E2-treated mice suggested that E2 was mainly implicated in Treg cell expansion during pregnancy (92). Again, they demonstrated that E2 reduces activation of effector T cells while promoting Treg cell function in mice (93). Prieto et al. also showed that E2 promotes human Treg cell proliferation and enhances their suppressive functions (94). By showing that Treg cell frequency and E2 levels are correlated in humans, Arruvito et al. corroborated the pro-Treg effect of E2 (35). This correlation between E2 and Treg cells was confirmed in mouse models by Tai et al., who demonstrated that E2 addition converts CD4+CD25− T cells in CD4+CD25+ Treg cells and enhances Foxp3 and IL-10 expression (95). Later, Valor et al. confirmed that E2 enhances Treg cell number and function in humans (96). Concerning Th17 cells, it has been shown for a long time that E2 suppresses experimental allergic encephalomyelitis (EAE), a Th17-mediated disease (97, 98), and E2 inhibits IL-17 production by murine lymphocytes (99). On the contrary, Khan et al. found that E2 promotes IL-17 production as well as RORγ expression in stimulated splenocytes in mice (100). Even if those studies revealed contradictory results, they suggest that E2 regulates Th17 cells, perhaps depending on the context. In EAE, the E2 protective effects seem to be due to Th17 inhibition (101). Recently, it has been also shown that E2 exerts inhibitory effects on Th17 cells in the bone environment (102). Ovariectomized mice have increased Th17 cells and associated factors in bone marrow, and this was reversed by E2 supplementation. So, it seems that E2 impact on Th17 depends on the tissue and disease context. However, most studies tend to show that E2 has an inhibitory role on Th17 cells. Concerning the impact of E2 on T cells, we can conclude that they are quite large. E2 can promote Th1, Th2, Treg, and Th17 cells depending on the context. E2 have also inhibitory impact on those cells. During pregnancy, it seems that E2 favors Th2 and Treg cell development while it dampens Th1 responses. There has been no study so far to explore the effect of E2 on Th17 during pregnancy. Concerning the molecular mechanisms implicated in the immunomodulatory roles of E2, a recent article has shown that those E2 effects are mediated through its receptors and involve intracellular signaling pathways like ERK, CREB, and Akt, as well as antioxidant enzymes (103).

### Human chorionic gonadotropin

**Figure 2:**
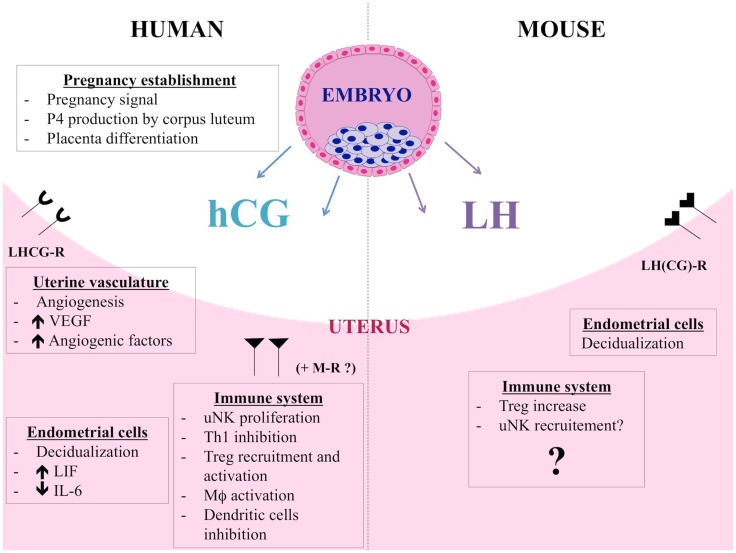
**hCG and LH functions during human and murine pregnancy**. Besides its endocrine role, hCG also acts on endometrial cells in a paracrine way and induces morphological and functional differentiation of endometrial stromal cells into decidua. hCG controls LIF and IL-6 secretion by human endometrial cells. By promoting angiogenesis, vasculogenesis, and angiogenic molecules production, hCG allows placenta to have adequate blood supply during the invasion of uterus and optimum nutrition to the fetus. The immunomodulatory properties of hCG are multiple. hCG has a positive impact on uNK cells, regulating their proliferation, putatively via mannose receptor (MR). Furthermore, hCG induces macrophage function, influences dendritic cell differentiation and function, and inhibits Th1 cells. Finally, hCG attracts Treg cells during early pregnancy and increases their frequency and suppressive activity. LH and hCG share the same LHCG receptor but LH is the only ligand of LHCG-R in mouse. hCG and LH are distinct molecules and actions of hCG cannot be claimed for LH. Murine blastocysts express the *Lh* gene and produce a bioactive LH signal thus showing that LH could be an important actor for the early dialog between the murine embryo and its mother. Data about the immunomodulatory roles of LH are scarce. LH could contribute to fetal tolerance by acting on murine Treg cells, similarly to hCG. LH would have also an impact on uNK cell recruitment.

Human chorionic gonadotropin is the most specific embryo-derived signal observed in humans and the *hCG* gene is transcribed as early as the eight-cell stage, before embryo implantation (104–107). This signal announces the presence of an embryo to the maternal organism. The hCG plasma level during human pregnancy is depicted in Figure 1. hCG belongs to the glycoprotein hormone family such as luteinizing hormone (LH), follicle-stimulating hormone (FSH), and thyroid stimulating hormone (TSH). Composed of two subunits, the alpha subunit is identical for all the members of the family while the hCG beta subunit shows 96% of homology with LH beta subunit. LH and hCG share the same LHCG receptor (LHCG-R). The basic endocrine function of hCG is to promote pregnancy via CL survival and stimulation of P4 production. Being released before embryo implantation, hCG also acts on endometrial cells in a paracrine way. For example, hCG induces morphological and functional differentiation of endometrial stromal cells into decidua (108). The investigators demonstrated that hCG induce prolactin secretion by human endometrial stromal cells, which is a sign of decidualization. Furthermore, hCG controls LIF and IL-6 secretion by human endometrial cells, and these two cytokines are known to influence blastocyst implantation (109). Indeed, we showed that endometrial epithelial cells cultured with hCG secrete higher LIF level while showing decreased IL-6 secretion. Furthermore, hCG has angiogenic and immunological properties, as reviewed in a recent paper (110). Briefly, hCG promotes angiogenesis by increasing vessel formation and pericyte sprouting and maturation in several *in vitro* and *in vivo* experimental models (111–115). hCG also influences angiogenic molecule production like vascular endothelial growth factor (VEGF) (113, 116). In this respect, hCG is considered to be an angiogenic factor (117). By promoting angiogenesis and vasculogenesis, hCG allows placenta to have adequate blood supply during the invasion of uterus and optimum nutrition to the fetus. The immunomodulatory properties of hCG are multiple and extremely important. First, hCG has a positive impact on uNK cells, the predominant leukocyte subtype of the gravid uterus that acts on establishment and maintenance of pregnancy, in humans and mice (2, 3, 118). Particularly, uNK cells contribute to essential vascular changes by regulating the remodeling of decidual spiral arterioles (119, 120) and by secreting angiogenic factors as members of VEGF family (121). It has been shown that hCG regulates uNK proliferation (122). A dose-dependant increase in uNK proliferation is observed when isolated human uNK are incubated with hCG (123). Since uNK cells do not express the LHCG-R (124), hCG would act on mannose receptor (MR), which is expressed by human uNK (123). Furthermore, hCG promotes monocyte function and their IL-8 production (125), and also induces macrophage functions (126). This promotes clearance of apoptotic cells and defense against infections, two relevant mechanisms for pregnancy maintenance. hCG influences also dendritic cell differentiation and function, decreasing their ability to stimulate T cell proliferation (127). Finally, hCG has different effects on CD4+ T cells. During the 1970s, hCG was suggested to have effects on maternal lymphocytes (128). Khan revealed that hCG treatment of NOD mice prevent them to develop diabetes, a Th1 disease. The investigators demonstrated that hCG injections in NOD mice before the onset of diabetes avoid the apparition of inflammatory infiltrate in pancreas and reduce hyperglycemia. They proved that hCG inhibits murine Th1 cells and their production of IFN-γ (129). A few years later, Khil confirmed those results and added new information. He showed that hCG effects on NOD mice implied an inhibition of T cell proliferative responses as well as an increase of CD4+CD25+ cells (130). He revealed that CD4+CD25+ depletion cancels the protective effects of hCG treatment on diabetes development. Recently, studies about hCG impact on Treg cells have been carried out. Schumacher et al. explored Treg cell recruitment at the human materno-fetal interface and demonstrated with migration assays that hCG attracts them during early pregnancy (131). The same group also showed that hCG increases murine Treg cell frequency *in vivo* and their suppressive activity *in vitro* (132). hCG treatment of abortion-prone female increases Treg cell numbers and reduces abortion rates. Thus, among its numerous functions during pregnancy affecting the fetus, placenta, and uterus (133), hCG has multiple immunomodulatory roles. In particular, the hCG effects on Treg and uNK cells, two major immune cell populations for pregnancy, demonstrate the importance of this embryo signal as an immune regulator during pregnancy. The different actions of hCG are summarized in Figure 2.

In humans, hCG shares its receptor with LH, a gonadotropin produced by the pituitary gland. Essential for reproduction, its fundamental functions are to initialize the final oocyte maturation and trigger ovulation during estrous cycle. LH controls P4 production by CL and if pregnancy occurs, hCG takes over the role of LH on P4 regulation. For pregnancy establishment, LH contributes to decidualization triggering. Mouse genome does not include a chorionic gonadotropin gene, and LH is therefore the only ligand of LHCG-R in mice. But hCG and LH are distinct molecules and actions of hCG cannot be claimed for LH (134). Data about the immunomodulatory roles of LH is scarce. Very recently, Schumacher has shown that LH could contribute to fetal tolerance by acting on murine Treg cells (135), following the example of hCG. Indeed, they showed that LH increases Treg cells peripherally and locally. They found also that LH injections reduce the abortion rates in abortion-prone mice. Earlier, we demonstrated that murine blastocyst express the *Lh* gene and produce a bioactive LH signal (136). Indeed, we detected *Lh* transcripts in blastocysts and the LH protein in their culture media. This LH is able to stimulate testosterone production by Leydig cells, and is thus bioactive. Furthermore, we detected *lhcgr* transcripts in mouse endometrium and the higher expression level was observed during the theoretical embryo implantation period. Thus, we suggested that LH could be an important actor for the early dialog between the murine embryo and its mother (136). The results of Schumacher et al. confirm this hypothesis. Furthermore, Van den Heuvel et al. explored adhesion properties of human lymphocytes in uterus and revealed that adhesion was increased under LH surge (137). The authors suggested that LH could activate adhesion molecules on the surface of uNK precursors, thereby enhancing their recruitment in the uterus. In their review, the same team puts forward the hypothesis that cyclic hormonal variation could generate a favorable period for uNK cell recruitment via expression of adhesion molecules. They suggest again that LH would have an impact on uNK cell recruitment (138). Altogether, those studies show that LH also exerts immunomodulatory roles. LH can regulate Treg cells and would act on recruitment of uNK cells. The study of immunological properties of LH is at the beginning but the expression of LH receptor by T lymphocytes (124) suggests that other functions could be explored. LH actions during murine gestation are summarized in Figure 2.

## Conclusion

Nowadays, uNK cells are considered as essential immune cells for pregnancy establishment and maintenance. CD4+ T cells are also important actors with Th2 being increased during pregnancy while Th1 have to be decreased for gestation to run smoothly. The importance of Treg cells for embryo implantation and pregnancy is also more and more evidenced. They are recruited before implantation to induce a favorable environment for embryo nidation. Afterwards, they are essential for maintenance of pregnancy. On the other hand, decreased number or less efficient Treg cells are implicated in fertility disorders. With regard to Th17 cells, the few available studies seem to indicate that they have a negative impact on fertility. Indeed, Th17 frequency is increased in infertile patients. Concerning immune properties of pregnancy hormones, it appears that they have positive impact on cells indispensable for implantation and gestation. With the combination of its pro-effects on Th2 and Treg cells, and anti-effects on Th1 and Th17 cells, P4 contributes clearly to establishment of favorable environment for pregnancy. E2 effects are more dependent on the context but it seems that E2 promotes Treg and Th2 cells while inhibiting Th1 cells.

Human chorionic gonadotropin positively influences the activities of Treg and uNK cells. This embryo signal is an essential actor for the success of pregnancy, both as the well-known endocrine factor regulating P4 secretion by the ovarian CL, but also as a paracrine agent during implantation as well as an angiogenic and immunologic mediator during the course of gestation. LH immune properties begin to be studied but its positive impact on Treg cells suggests that LH could be a considerable immunomodulator. The timing of pregnancy hormones and CD4+ T cells levels during human pregnancy is simplified on Figure 1. Of course, endocrine system is not the only factor responsible for immune cell recruitment and activation during pregnancy. It was showed that Treg cells found during pregnancy react to paternal antigen (22, 24). Zhao et al. demonstrated that fetal alloantigens are responsible for Treg cell recruitment, excluding hormonal influence (139). To finally conclude, both allogeneic and hormonal stimulation are responsible for a harmonious regulation of the immune system leading to a successful pregnancy.

## Conflict of Interest Statement

The authors declare that the research was conducted in the absence of any commercial or financial relationships that could be construed as a potential conflict of interest.
